# Micropropagation and Genetic Fidelity of Fegra Fig (*Ficus palmata* Forssk.) and Grafting Compatibility of the Regenerated Plants with *Ficus carica*

**DOI:** 10.3390/plants13091278

**Published:** 2024-05-06

**Authors:** Ahmed Ali Al-Aizari, Yaser Hassan Dewir, Abdel-Halim Ghazy, Abdullah Al-Doss, Rashid Sultan Al-Obeed

**Affiliations:** Plant Production Department, College of Food and Agricultural Sciences, King Saud University, Riyadh 11451, Saudi Arabia

**Keywords:** acclimatization, dark incubation, in vitro propagation, molecular markers Moraceae, polymorphism

## Abstract

*Ficus palmata* is an important fig species that produces edible and nutritious fruit and possesses several therapeutic uses. This study reports an effective method for the micropropagation of *F. palmata* using nodal explants. In vitro shoots were cultured for 7 weeks onto MS medium fortified with different concentrations of cytokinins, light intensities, sucrose concentrations, and light/dark incubation treatments. Optimal axillary shoot proliferation (10.9 shoots per explant) was obtained on a medium containing 30 g/L sucrose and supplemented with 2 mg/L 6-benzylaminopurine (BAP) under 35 μmol/m^2^/s light intensity. Dark incubation limited the foliage growth but favored shoot elongation and rooting compared with light incubation. Elongated shoots, under dark conditions, were rooted (100%; 6.67 roots per explant) onto MS medium containing 1 mg/L indole-3-acetic acid (IAA) and 1.5 g/L activated charcoal. The micropropagated plantlets were acclimatized with a 95% survival rate. In this study, the genetic fidelity of micropropagated *F. palmata* clones along with their mother plant was tested using randomly amplified polymorphic DNA (RAPD), inter-simple sequence repeats (ISSR), and start codon targeted (SCoT) molecular markers. The genetic similarity between the micropropagated plantlets and the mother plant of *F. palmata* was nearly 95.9%, assuring high uniformity and true-to-type regenerated plants. Using micropropagated *F. palmata* plantlets as a rootstock proved appropriate for the grafting *F. carica* ‘Brown Turkey’. These findings contribute to the commercial propagation and production of the fig crop.

## 1. Introduction

The genus *Ficus* belongs to the large family Moraceae and includes 876 accepted plant species (WFO Plant List, 2024; https://wfoplantlist.org/taxon/wfo-4000014727-2023-12?page=1; Accessed on 5 March 2024). In Saudi Arabia, there are five grown species of fig namely *Ficus vasta*, *Ficus carica*, *Ficus salicifolia*, *Ficus palmata*, and *Ficus glumosa* [1]. Fig propagation can be achieved through both vegetative and sexual methods. However, sexual propagation is not preferred because of the low seed viability. Due to the heterozygosity in sexual reproduction, this method is limited to breeding programs [2]. Vegetative propagation through cutting, grafting, and layering is traditionally used to propagate fig plant species [3]. For *Ficus carica* cultivation, own-rooted nursery stocks are widely used for planting. However, soil disorders pose serious threats to cultivation; as a result, rootstocks that are resistant to such soil disorders have long been desired. Additionally, for large-scale traditional propagation, several cuttings are needed to increase the cultivated area of figs. 

Introducing wild fig species as a rootstock for common fig trees would enable high resistance to diseases, e.g., Ceratocystis Canker [4]. Fegra fig (*Ficus palmata*) is a small, wild fig tree widely distributed in Nepal, Somalia, Egypt, India, Iran, Sudan, Ethiopia, and Saudi Arabia [5,6]. *Ficus palmata* fruit is a very nourishing food and is used for making various products [7]. The tree is used as fuel wood and is traditionally used for the effective treatment of many diseases, namely, skin diseases, ringworm, wound infections, and hemorrhoids [8]. Phytochemical analysis of the aerial parts of *F. palmata* has revealed several pharmacological activities [9,10,11,12,13,14]. Moreover, *F. palmata* has been reported as a potential rootstock for grafting *Ficus carica* ‘Masui Dauphine’ [15]. In vitro techniques have been utilized to achieve rapid mass propagation over conventional methods of vegetative propagation. Moreover, it ensures the production of true-to-type plants, uniform quality, and production of disease-free planting materials with seasonal independence [16]. Additionally, in vitro culturing can rejuvenate mature fig plantlets [17]. Several studies have reported the micropropagation of *Ficus carica* [18,19]. To our knowledge, in vitro propagation studies on *F. palmata* are limited to a study by El-Tarras et al. [20]; however, a low multiplication rate was reported. 

Several factors may significantly affect shoot multiplication in tissue cultures. One of the factors that affect in vitro plant regeneration is the presence of plant growth regulators (PGRs) in the culture media [21,22]. Shoot proliferation is mainly regulated by cytokinins. Several studies have been carried out to study the effectiveness of cytokinins on the multiple-shoot formation of different fig cultivars [19,23,24]. In addition, it is well established that sucrose in the medium plays an important role as a source of energy and a carbon compound for the growth and development of explants. The sucrose affects the regulation of growth genes so that the absence of sucrose can inhibit plant growth [25]. A few studies investigated the effects of sucrose on shoot multiplication of fig [26,27]. Also, light intensity is an important parameter that influences shoot regeneration, fresh weight, and secondary metabolite biosynthesis during micropropagation. Light can also influence the efficacy of PGRs as well as the adjustment of endogenous hormone levels [28]. Moreover, dark incubation has been reported to influence shoot regeneration in vitro. Incubation in the dark may delay the degradation of endogenous and/or exogenous plant growth regulators and reduce cell wall thickness, thus facilitating the translocation of plant growth regulators [29] and in turn regulating the morphogenic responses. However, to our knowledge, no studies have been conducted on the effect of duration and intensity of lighting on shoot multiplication and elongation of figs. 

The significance of tissue culture-based propagation techniques resides in the fact that the regenerated plant population should be genetically and morphologically uniform with donor mother plants [30]. The in vitro development of plants is governed by various factors, i.e., PGRs and light intensity [31,32]. Therefore, micropropagation may result in DNA sequence variation and the generation of somaclonal variation [33]. To overcome this, it is a prerequisite to validate the genetic homogeneity in early times to authenticate the quality of micropropagated plants for commercial use, particularly for woody plants [34]. The genetic homogeneity amongst the micropropagated plants with the mother plant has been assessed by PCR-based molecular markers, i.e., Amplified Fragment Length Polymorphism (AFLP), Inter Simple Sequence Repeats (ISSR), Random amplified polymorphic DNA (RAPD), Start codon targeted (SCoT), and Simple Sequence Repeats (SSR) [35,36]. RAPD, ISSR, and SCoT marker techniques have been successfully used to assess the genetic fidelity in several plants such as *Pittosporum eriocarpum* [37], *Rauwolfia tetraphylla* [38], and *Solanum khasianum* Clarke [39]. This study aimed to establish an efficient micropropagation of *F. palmata* as a rootstock for the common fig and a medicinal plant, using nodal explants and to assess the genetic fidelity of the micropropagated plants through RAPD, ISSR, and SCoT molecular markers.

## 2. Results and Discussion

### 2.1. Surface Disinfection and Culture Initiation of F. palmata

**Figure 1:**
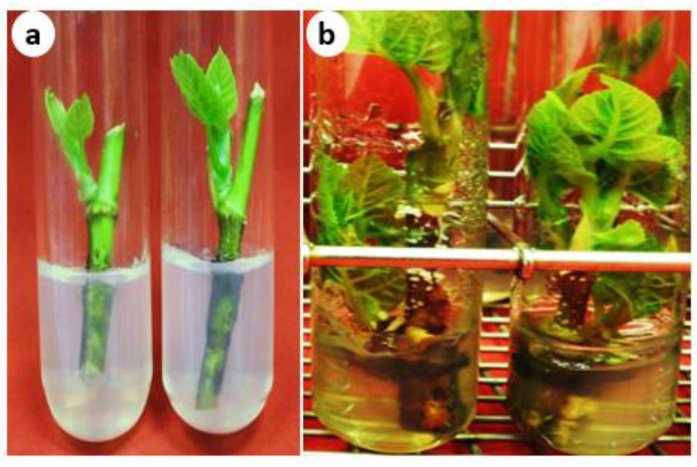
The establishment of an in vitro aseptic culture of *Ficus palmata*. (**a**) Nodal explants that had been soaked in antioxidant solution (100 mg/L citric acid and 150 mg/L ascorbic acid) prior to surface sterilization and (**b**) Nodal explants without soaking in antioxidant solution showing phenolic exudates.

Contamination was one of the major challenges faced during the aseptic culture establishment of *F. palmata*. The in vitro nodal culture of *F. palmata* was established with a 64.8% aseptic rate. Additionally, tissue browning is a common problem during the establishment of trees in vitro cultures. Soaking *F. palmata* nodal explants in citric acid and ascorbic acid solution prevented the occurrence of phenolic substances and tissue browning (Figure 1a) as compared with non-soaked explants (Figure 1b). Sodium hypochlorite (NaOCl) and mercuric chloride (HgCl_2)_ are commonly used surface disinfectants in plant tissue culture. El-Tarras et al. [20] reported a successful surface sterilization of *F. palmata* nodal explants using 70% ethanol for 1 min followed by 20% sodium hypochlorite solution (NaOCl; 5.25%) for 10 min, but the contamination percentage was not mentioned. Abdolinejad et al. [40] sterilized the stem segments of *F. carica* cultivars ‘Sabz’ and ‘Torsh’ using 15% NaOCl solution (containing 5% active chlorine) for 20 min. In our study, we found that using HgCl_2_ treatment was very effective for controlling microbial contamination of *F. palmata.* Prabhuling et al. [41] reported a successful surface sterilization of *F. carica* ‘Brown Turkey’ nodal explants using 2% NaOCl for 15 min followed by HgCl_2_ 0.1% for 3, 5, and 10 min. The maximum aseptic culture establishment (71%) was obtained when double nodal explants were treated with 0.1% HgCl_2_ for 10 min. Vanmathi et al. [42] also reported that treatment of nodal explants of *F. carica* with HgCl_2_ (0.1%) for 3 min was the most effective surface sterilization procedure for aseptic culture establishment (72%).

### 2.2. Effect of Cytokinin Concentrations on Axillary Shoot Multiplication

**Figure 2:**
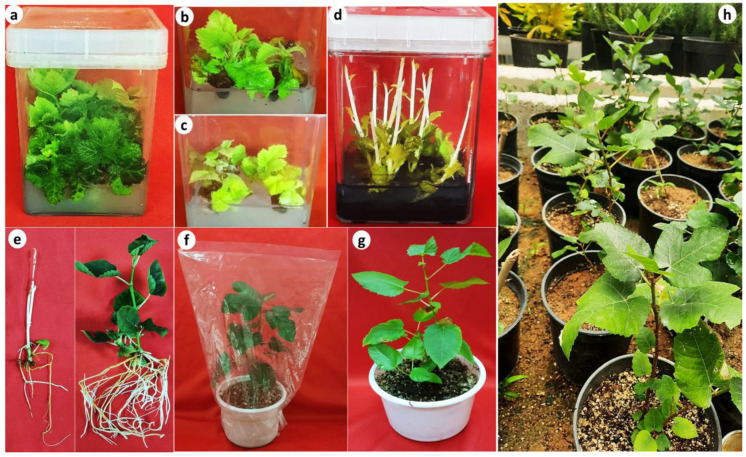
Micropropagation of the wild fig (*Ficus palmata*). (**a**) Axillary shoot multiplication using nodal explants cultured onto MS medium supplemented with 2 mg/L BAP after 7 weeks in culture, (**b**,**c**) negative effects of high sucrose (60 g/L) and high light intensity (70 PPFD), respectively, on shoot multiplication and growth, (**d**) elongation of individual axillary shoots cultured onto MS medium supplemented with 1 g/L activated charcoal under dark incubation, (**e**) in vitro rooting of axillary shoots onto MS medium supplemented with 1 mg/L IAA after 4 weeks of dark condition (left) and followed by 3 weeks under light incubation (right), (**f**) micropropagated plantlet covered with a polyethylene bag, (**g**) micropropagated plantlet following 4 weeks of acclimatization, and (**h**) micropropagated plants in the greenhouse after 12 weeks of acclimatization.

Both cytokinin types, their concentration treatments, as well as their interaction effects had significant effects (*p* < 0.05) on the number of axillary shoots, length of the longest shoot, fresh weight, and dry weight per explant (Table 1). Shoot induction was visible after two weeks of culture. When comparing the different types of cytokinins, BAP was found to be the most effective in the induction of multiple shoots, followed by thidiazuron (TDZ) and zeatin. The number of induced axillary buds increased with increasing BAP concentrations up to 2 mg/L and then decreased at 3 mg/L BAP. The treatment of (2.0 mg/L) BAP generated the highest number of axillary shoots with an average of 11.2 shoots per explant with an average length of the longest shoot of 1.9 cm (Table 1 and Figure 2a). The average fresh weight per explant was 3.64 g and the average dry weight per explant was 0.389 g in this treatment. Despite the high number of axillary shoots (9.8 shoots per explant) obtained with TDZ, it led to vitrification and an abundance of stunted leaves, especially at high concentrations. Among the different cytokinins tested, zeatin yielded the lowest number of axillary shoots (4.8 shoots per explant) (Table 1). In general, all cytokinin treatments resulted in higher axillary shoot multiplication and growth of explants than the control treatment. 

Cytokinin is well-known for its ability to promote shoot multiplication by acting as a signaling molecule involved directly in the cell division of meristematic cells [43]. They regulate apical dominance, the size of the shoot meristem, the amount of leaf primordia, shoot growth, leaf production, and the development of axillary buds [44]. However, the response of tissues towards exogenous plant growth regulators can be due to the interaction between the endogenous regulators or the physiological status of the plant, resulting in a different response depending on the growth regulator supplemented. In the present study, BAP was the most effective cytokinin in stimulating multiple shoot production of *F. palmata.* Furthermore, BAP’s slower rate of metabolism makes it more stable in tissue culture compared to other cytokinins [19]. The results in this study corroborate the previous finding of Ling et al. [19], whereby the maximum number of shoots (15.2 shoots per explant) were produced by the ’Violette de Sollies‘ fig cultivar when supplemented with 5.0 mg/L BAP as compared with other cytokinins (kinetin, TDZ, and zeatin). In addition, BAP at 0.4 mg/L was found to be the most effective cytokinin compared to kinetin and zeatin at 0.0–2.0 mg/L shoot multiplication (3.2 shoots per explants) of *Ficus carica* ‘Salti Kodari’ [23]. Furthermore, BAP treatments increased the number of multiple shoots induced in the shoot-tip explants of *F. carica* ‘Japanese BTM 6’ when compared to zeatin [45]. For *F. palmata*, it has been reported that 2.0 mg/L BAP produced the maximum number of multiple shoots (3.25) using nodal explants [20]. Shoot multiplication is influenced by several factors, i.e., the physiological state of the mother plant, culture conditions and period, type and number of explants in a culture vessel, and size of the culture vessel. El-Taras et al. [20] did not clearly state these conditions in their research; hence, the obtained low multiplication rate (3.25) despite using the same BAP concentration (2 mg/L) could have been attributed to variable conditions compared to our study. In general, BAP proved more effective than TDZ and zeatin for axillary shoot multiplication of *F. palmata*.

### 2.3. Effect of Light Intensity and Sucrose Concentration on Axillary Shoot Multiplication

The effects of light intensity and different concentrations of sucrose on shoot multiplication were evaluated in the optimized medium (MS containing 2 mg/L BAP). Both light intensity and sucrose concentration treatments, as well as their interaction, had significant effects (*p* < 0.01 and *p* < 0.001) on the number of shoots, length of the main shoot, fresh weight, and dry weight of *F. palmata* shoots. For the light intensity in dry weight, there was no significant difference (Table 2). The treatment of 35 μmol m^−2^∙s^−1^ PPFD and 30 g/L sucrose was optimal for shoot multiplication of *F. palmata* and generated the highest number of axillary shoots (10.29 shoots per explant) with an average length of the main shoot of 6.7 cm. The average fresh weight per explant was 3.7 g and the average dry weight per explant was 0.34 g (Figure 2 and Table 2). 

It is well-established that light intensity affects plant growth and shoot multiplication in vitro. Nevertheless, studies on the effect of light intensity on shoot multiplication of fig in vitro cultures are lacking. However, plant responses to varying light intensities are species-specific. High light intensity at 150 μmol m^−2^∙s^−1^ was the optimal level for the shoot multiplication of *Castanea sativa* [46]. Conversely, low light intensity at 25 μmol m^−2^∙s^−1^ light intensity was optimal for the shoot multiplication of *Philodendron bipinnatifidum* [47]. De Riek et al. [48] highlighted the importance of sucrose for culture growth and biomass increase. In our study, all parameters decreased with increasing light intensity and sugar concentration; the plantlets showed slower growth and yellowing of the leaves was observed particularly in the treatment 60 PPFD and 60 g/L sucrose. The optimal sucrose concentration for *F. palmata* multiplication was 30 g/L. Elazab and Shaaban [26] stated that the optimal treatment for the proliferation of *F. carica* was 30 g/L sucrose, and a high concentration of sucrose reduced the number of shoots, the length of the shoots, the number of leaves on each shoot, and the area of the leaves. These negative effects could be due to the decrease in the osmotic capacity of cells and their exposure to stress [49,50]. Higher concentrations of sucrose also lead to an increase in the concentration of detrimental phenolic compounds and inhibition of the growth of plant tissues [51,52]. In addition, it reduces leaf pigments and limits the efficiency of the photosynthesis system [53]. Conversely, a high concentration of sucrose in the plant tissue culture system may have a positive effect on the growth and development of micropropagated plants, such as *Ficus carica*, in which 45 g/L sucrose treatments resulted in the highest shoot fresh and dry weight [27]. However, plant responses to sucrose are species-specific. Cao et al. [54] reported that sucrose concentrations above 2% decreased *Vaccinium corymbosum* ‘Duke’ shoot proliferation. Similarly, sucrose concentrations above 2% inhibited the shoot growth of *Dactylorhiza majalis* and *Dactylorhiza incarnata* ssp. incarnata [55].

### 2.4. The Effect of Dark Incubation versus Standard Photoperiod Conditions on the Growth and Elongation of Axillary Shoots

**Figure 3:**
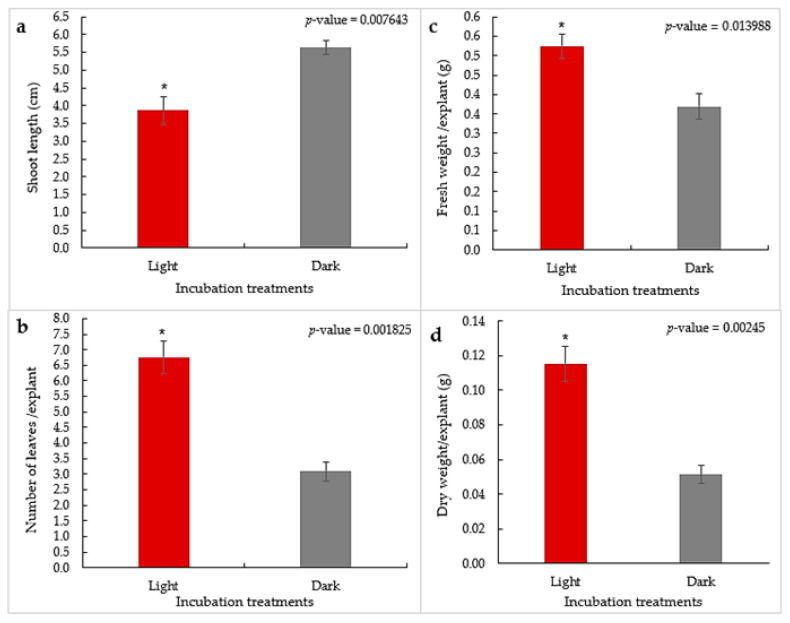
Shoot length (**a**), number of leaves (**b**), fresh weight (**c**), and dry weight (**d**) of *F. palmata* axillary shoots as influenced by light and dark incubation treatments after 4 weeks in culture. (Bars represent means ± standard error, * = significantly different at *p* ≤ 0.05 according to Student’s unpaired *t*-test).

The treatment of dark incubation versus standard photoperiod conditions (16 h photoperiod at 35 μmol m^−2^·s^−1^) had significant effects (*p* ≤ 0.05) on the length of shoots, number of leaves, shoot fresh weights, and shoot dry weights (Figure 3a–d). Shoots grown under dark incubation showed the highest shoot length (5.63 cm) compared with plantlets grown under light (3.87 cm) after 4 weeks of culture. Conversely, plantlets grown in the light treatment showed the highest average number of leaves (6.75) with the highest fresh weight (0.524 g) and dry weight (0.116 g). compared with shoots grown under dark conditions. Our findings stressed the important influence of darkness on the elongation of *F. palmata* axillary shoots. Light and darkness both act as environmental cues that regulate plant growth and development. Darkness leads to shoot elongation by modulating the levels of phytohormones [56]. *Ficus palmata* shoots grown in the dark facilitated shoot elongation as compared to those growing under the standard light regime, which was favorable for the next micropropagation step (rooting). Similar results were obtained in other plant species, such as *Catasetum fimbriatum* [57,58]. Light enhances plant morphology designed to carry out photosynthesis, whereas darkness leads to fewer leaves and increases cell elongation [59]. 

### 2.5. Effect of Auxins Concentrations on In Vitro Rooting of F. palmata Axillary Shoots

The type of auxin had a significant effect on all parameters except for the number of roots. Auxin concentrations have a significant effect (*p* < 0.05) on the rooting percentage, number of roots, and root length while the interaction between auxin type and auxin concentrations had no significant effects on rooting parameters (Table 3). Root induction began after 2 to 3 weeks of incubation from the cut ends of the microshoots in most cultures tested. When comparing the auxins tested, IAA proved more effective for in vitro rooting of *F. palmata* than IBA and NAA. The highest rooting percentage (75%) and length of the main root (3.9 cm) were obtained on medium supplemented with 1 mg/L IAA. A low concentration of NAA (0.1 mg/L) resulted in the highest number of roots (2.3 roots per explant) while the highest fresh and dry biomass were obtained at 1 mg/L IAA. High concentrations of auxins at 2 mg/L negatively affected the in vitro rooting and growth of *F. palmata* axillary shoots. Rooting the plantlets is one of the most crucial stages of micropropagation and is a prerequisite for successful acclimatization. The effects of auxin type and concentration on root formation depend on the carry-over effects of cytokinin during the multiplication stage and vary considerably among different plant species [60]. Additionally, possible changes in affinities for auxin receptors and variances in uptake, transport, and metabolism are other factors contributing to the different effectiveness observed among the three auxins [61]. Parab et al. [24] concluded that IAA was better than IBA in rooting the *Ficus carica* ‘Black Jack’. Ling et al. [62] found that IAA was the best auxin for *Orthosiphon stamineus* in adventitious root formation compared with IBA and NAA. 

IBA was used as a prime auxin that produced the highest root formation and number of roots on different cultivars of fig (*Ficus carica* L.) [19,23,41,63], and proved effective for in vitro rooting of several woody plant species such as Cancer bush (*Lessertia frutescens*) [64], water berry tree (*Syzygium cordatum*) [65], and buttonwood tree (*Conocarpus erectus*) [66]. The ability of IBA to stimulate root growth can be attributed to its relatively high stability. In addition, IBA functions as a hormone with a slow-release mechanism, allowing its effects to persist for a longer duration in the culture media. This may lead to continuous stimulation of root growth [67]; however, the continuous presence of IBA in the medium may be inhibitory for root elongation during the rooting phase [68]. It has the capacity to activate the gene accountable for root formation [69]. IBA can also be converted into free IAA within the medium, hence facilitating a consistent presence of IAA without the need for direct IAA supplementation for plant regeneration [70]. Conversely, the low absorption efficiency of NAA makes it unsuitable for the regeneration of some plant tissues, as it could potentially lower rooting efficiency in culture [71]. In addition, it leads to callus development at the end cut of microshoots of *Hebe buchananii, Hebe canterburiensis* [72], and *Ruta chalepensis* [73]. However, NAA showed efficacy in stimulating root formation in vitro in clones of *Ficus carica*. It was observed that NAA effectively stimulated the highest average number of roots (4.87) and length (4.07 cm) for *Ficus carica* ‘Sultani’ explants [17]. In general, the formation of roots differed significantly depending on the type of auxin and its concentration in the cultured medium during the rooting stage [74].

### 2.6. Effect of Initial Dark Incubation versus Standard Photoperiod Conditions on In Vitro Rooting of F. palmata Microshoots

The implementation of a dark phase as a first step during the rooting stage had a significant effect (*p* < 0.05) on all rooting parameters (rooting percentage, number of roots, root length, fresh weight, and dry weight) while activated charcoal and their interaction showed no significant effects (Table 4). Incubation in the dark resulted in 100% rooting of microshoots and favored a higher number of roots per explant (6.67) when the medium was supplemented with activated charcoal as compared with other treatments. Conversely, under light incubation, the plantlets exhibited higher values of root length and fresh and dry weights (Table 4). An incubation period of darkness at the beginning of the rooting process, with exposure to an auxin, has been shown to be beneficial for the rooting of many woody plant species [75], and this may be attributed to natural auxin enhancement under dark conditions. Additionally, dark incubation may potentially impact the various stages of auxin metabolism, particularly regarding root development, by modifying the activities of peroxidase and endogenous phenolic compounds [76]. Furthermore, research has demonstrated that incubating *Petunia hybrida* cuttings in the darkness increases the concentration of naturally occurring auxin at the base of the stem [77]. Dark incubation treatment also enhances the allocation of carbohydrate resources toward the zone where roots are formed [78], thus enhancing the energy resources required for the development of the new organ. 

### 2.7. Acclimatization

Healthy regenerated plantlets of *F. palmata* with 6–8 leaves and a well-developed root system (Figure 2e, right) were successfully hardened in pots containing a sterile mixture of peatmoss and perlite (1:1; *v/v*) in a growth chamber before being transplanted ex vitro. After 4 weeks, the acclimatized plants were transferred to pots with a mixture of soil and peatmoss (1:1; *v/v*), and 95% of the plantlets survived when transferred to greenhouse conditions. Previous studies on acclimatization of micropropagated *Ficus carica* plants reported 80% survival in a mixture of soil, perlite, and peat (1:1:1; *v/v/v*) [23], 90.25% survival in coco peat [63], and 100% survival in a biochar soil [19].

### 2.8. Assessment of Genetic Fidelity Using RAPD, ISSR, and SCoT Molecular Markers

**Figure 4:**
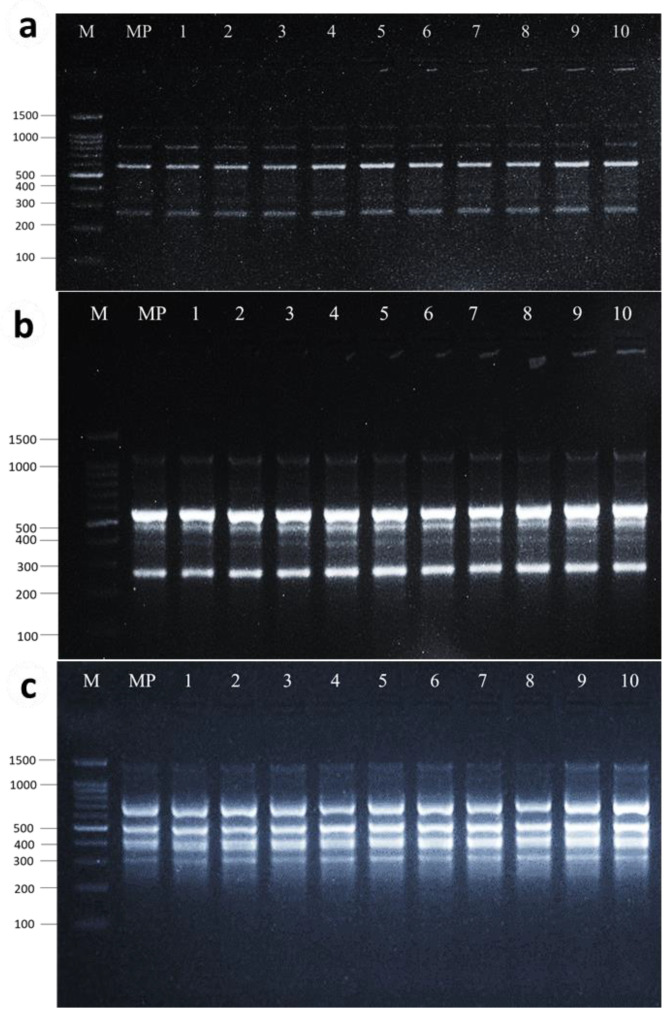
DNA amplification pattern obtained with the RAPD primer (**a**), ISSR primer (**b**), and SCoT primer (**c**). Lane M = DNA ladder; Lane-MP = DNA from mother plant; Lane 1–10 DNA from micropropagated plants.

The genetic fidelity of micropropagated *F. palmata* plantlets was assessed using RAPD, ISSR, and SCoT molecular markers in ten plants randomly selected along with the mother plant. A total of 102 bands were obtained from the three markers in which only four bands were polymorphic representing 3.9% polymorphism. In RAPD analysis, among ten RAPD primers screened, eight primers produced 22 clear, reproducible bands, and the number of bands produced by the eight primers ranged between 1 and 4 with an average of 2.75 bands/primer with band size (in bp) ranging from 200 to 1500 (Table 5, Figure 4). All bands were monomorphic among the tested plants and the mother plant. In ISSR analysis, ten primers produced 50 bands which were clear, distinct, and scorable with 4% polymorphism (2 bands), while all the other bands produced were monomorphic. The number of bands produced by the ten primers ranged between 2 and 8 with an average of 5 bands/primer with a band size (in bp) ranging from 200 to 1500 (Table 5, Figure 4). In the SCoT analysis, among the ten SCoT primers screened, eight primers produced 30 bands which were clear, distinct, and scorable with 6.6% polymorphism (2 bands), while all the other bands produced were monomorphic. The number of bands produced by the eight primers ranged between 1 and 6 with an average of 3.75 bands/primer with a band size (in bp) ranging from 250 to 1500 (Table 5, Figure 4). The retention of true-to-type in in vitro regenerated plants is critical for any micropropagation system, as well as for conservation programs. Genetic variations could occur in tissue culture-regenerated plants [79]. The development of somaclonal anomalies between the regenerated plants may limit the effectiveness of the micropropagation protocol [80,81]. Various factors such as genotype, explant type, culture periods, and growth regulator combinations and concentrations may disturb the internal polarity and physiology of the explants [82,83], and could also influence the true-to-type of tissue cultured plants [83,84]. Therefore, evaluating the genetic integrity of regenerated plants is critical. PCR-based markers are one of the most significant approaches being used to test genetic fidelity in several plant species. For better analysis of genetic fidelity, using more than one marker was always recommended [85]. Additionally, Palombi and Damiano [86] suggested the use of more than one DNA amplification technique as advantageous in evaluating somaclonal variations in micro-propagated plants of kiwi fruit. In the present study, no major differences between *Ficus palmata* micropropagated plants and the mother plant were found. The high ratio of monomorphic banding pattern in the micro-propagated and mother plant and the low frequency of polymorphism in ISSR and SCoT profile of micropropagated plantlets (4.0 and 6.6, respectively) revealed a high rate (95.9%) of genetic stability, but also a certain (4.1%) level of somaclonal variation. A previous study by Dessoky et al. [34] reported a < 6% polymorphism frequency using RAPD and ISSR molecular markers of micropropagated fig (*Ficus carica*) regenerated via axillary shoot multiplication. 

Many reports in the literature suggest that plants regenerated through organized tissues such as axillary shoots maintain the genetic integrity of the plantlets [34,87,88,89]. This further supports the fact that axillary multiplication is the safest mode of micro-propagation to produce true-to-type progeny. The RAPD method was sensitive and capable of detecting variations in plant genome profiles [90,91] and regenerated plantlets [92]. In our study, along with the RAPD primer, we also used other advanced primers, i.e., ISSR and SCoT, which are reliable and reproducible types in assessing the genetic fidelity of micropropagated plants compared to RAPD [37]. Among the various markers, SCoT markers are gaining much attention due to their superiority over other markers [93,94]. They can be used effectively for both genetic diversity and studies of genetic fidelity [95,96]. SCoT markers have been reported for assessing the genetic fidelity of several plant species such as *Cleome gynandra* [97], *Bauhinia racemosa* [81], and *Rauwolfia tetraphylla* [38]. Similarly, the use of RAPD and ISSR markers for assessing the genetic fidelity of *Ficus carica* micropropagated plants was reported by several studies [34,40,87,98]. However, this is the first report using RAPD, ISSR, and SCoT primers for the assessment of the genetic fidelity of micropropagated *F. palmata* plants.

### 2.9. Grafting Compatibility of Micropropagated F. palmata Plantlets as a Rootstock for F. carica ‘Brown Turkey’

**Figure 5:**
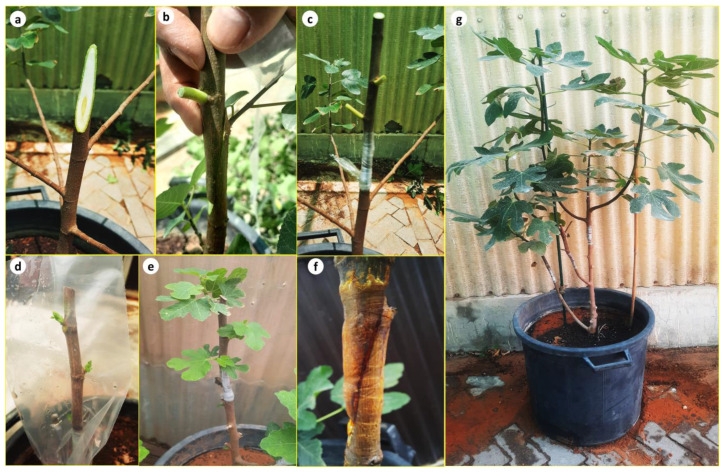
Splice grafting between micropropagated *Ficus palmata* as a rootstock and *Ficus carica* ‘Brown Turkey’ as a scion. (**a**) Front view of the rootstock cut surface, (**b**) placing the scion on rootstock, (**c**) tying the scion and rootstock together with grafting tape, (**d**,**e**) scion growth on the rootstock after 3 and 8 weeks of grafting, respectively (**f**), side view of the grafted area showing junction between the scion and rootstock after 12 weeks of grafting, and (**g**) scion growth on the rootstock after 7 months of grafting.

Micropropagated *F. palmata* plantlets proved to be a compatible rootstock for *F. carica* ‘Brown Turkey’ using splice grafting (Figure 5a–c). Scion growth was observed within 3 weeks of grafting and full junction was observed at 12 weeks (Figure 5d–f) with a 100% survival rate. A previous study by Hosomi [15] investigated the graft compatibility between rootstocks of wild *Ficus* species and scions of fig cultivar ‘Masui Dauphine’ and the best compatibility was observed with *F. palmata*. The grafting technique is widely used for the production of fruit trees either to overcome soil disorders or to improve quality and productivity. However, further studies on the effect of using *F. palmata* rootstock on the growth and productivity of the fig scions are required.

## 3. Materials and Methods

### 3.1. Plant Material, Surface Sterilization, and Establishment of Aseptic Culture 

**Figure 6:**
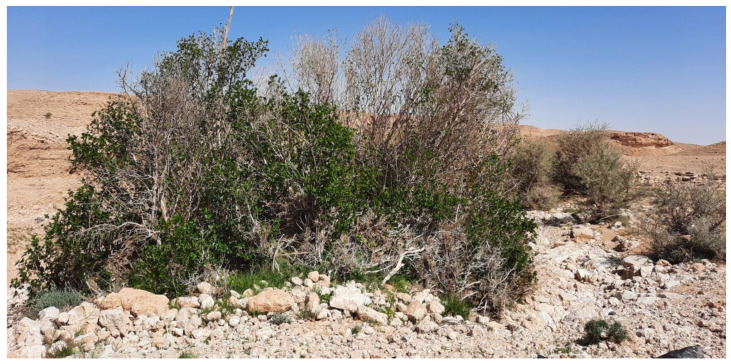
*Ficus palmata* mother tree located in the Huraymila district, Saudi Arabia.

Twigs of *F. palmata* Forssk. (20–30 cm in length) were collected in January 2020 from one mature tree located in Huraymila district (25°7′36″ North, 46°7′21″ East), Saudi Arabia (Figure 6). Nodal segments (2–4 cm) were excised from the twigs and washed under running tap water for 30 min to remove the superficial dust followed by a detergent for 3 min. The explants were dipped in a solution of 100 mg/L citric acid and 150 mg/L ascorbic acid for 20 min, to avoid browning of tissues, followed by surface sterilization in 70% ethanol for 1 min. Then, they were rinsed in 0.1% HgCl_2_ for 8 min, followed by 40% Clorox^®^ solution (5.25% NaOCl; National Cleaning Products Co. Ltd., Dammam, Saudi Arabia) containing 2 drops of Tween-20 for 15 min. Finally, the explants were washed with sterile distilled water 4–5 times. The explants were cultured in glass test tubes (24 mm × 200 mm) containing 15 mL MS medium [99] supplemented with 2 mg/L 6-benzylaminopurine (BAP) and 0.1 mg/L IBA, 3% sucrose and 0.8% agar. After four weeks of incubation, observations on percentages of contamination were recorded.

### 3.2. Standard Medium Preparation and Growth Conditions 

The pH of the medium was adjusted to 5.8 before autoclaving (at 121 °C and 1.2 kg cm^−2^ pressure for 15 min). The cultures were incubated at 25 ± 2 °C under a 16 h photoperiod provided by cool white fluorescent lights at 35 μmol m^−2^·s^−1^ photosynthetic photon flux density (PPFD) and a relative humidity of 50–60%.

### 3.3. The Effect of Cytokinin Concentrations on Axillary Shoot Multiplication

Nodal explants (1.5 to 2 cm length, with a single bud) were inoculated for 5 weeks to Magenta vessels (77 mm × 77 mm × 97 mm; Sigma Chemical Co., St. Louis, MO, USA) containing 60 mL MS medium, 3% sucrose, and 0.8% agar and supplemented with various concentrations of BAP (0.1, 1.0, 2.0, 3.0 mg/L), TDZ, or zeatin (Sigma Chemical Co., St. Louis, MO, USA) (0.1, 0.5, 1.0, 1.5 mg/L). The cultures were then inoculated onto MS medium without plant growth regulator (PGRs) for 4 weeks and kept under the same conditions. There were four replicates in each treatment and each replicate was represented by a Magenta vessel containing 4 nodal explants. After seven weeks of incubation, observations on the number of axillary shoots per explant, length of the longest shoot per explant, shoot fresh weight, and shoot dry weight were recorded. Dry weight was measured after drying the shoots for 48 h at 60 °C.

### 3.4. The Effect of Sucrose Concentrations and Light Intensity on Axillary Shoot Multiplication

Nodal explants (4 per culture vessel) were inoculated to magenta vessels containing 60 mL MS medium with varied concentrations of sucrose (1.5, 3.0, 4.5, 6.0%). All media were supplemented with 2 mg/L BAP as the optimal cytokinin concentration and gelled with 0.8% agar. The cultures were incubated for 5 weeks at 25 ± 2 °C under a 16 h photoperiod provided by cool white fluorescent lights at (15, 35, 70 μmol m^−2^·s^−1^) PPFD, followed by 4 weeks culture onto MS medium without PGRs and kept under the same conditions. After nine weeks of incubation, observations on the number of axillary shoots, shoot length, shoot fresh weight, and shoot dry weight were recorded. 

### 3.5. The Effect of Dark Incubation versus Standard Photoperiod Conditions on the Growth and Elongation of Axillary Shoots 

Axillary shoot clumps (4 per culture vessel) were inoculated onto Magenta vessels containing 60 mL MS medium containing 3% sugar and supplemented with 1.5 mg/L activated charcoal and 3 mg/L silver nitrate for their elongation. The cultures were incubated for 4 weeks at 25 ± 2 °C under dark or a 16 h photoperiod provided by cool-white fluorescent lights at 35 μmol m^−2^·s^−1^ PPFD. After four weeks of incubation, observations on the shoot length, number of leaves, shoot fresh weight, and shoot dry weight were recorded. 

### 3.6. The Effect of Auxins Concentrations on In Vitro Rooting of F. palmata Axillary Shoots

Elongated axillary shoots (4–5 cm) were individually separated and cultured onto MS medium containing 3% sucrose and supplemented with varied concentrations of IBA, NAA, and IAA (Sigma Chemical Co., St. Louis, MO, USA) at 0.1, 0.5, 1.0, 2.0 mg/L. All cultures were incubated for 7 weeks under standard growth conditions as described earlier. After seven weeks in culture, the percentage of rooted plants, the number of roots per plant, length of roots, and fresh and dry weights were recorded. 

### 3.7. The Effect of Initial Dark Incubation versus Standard Photoperiod Conditions on In Vitro Rooting of F. palmata Axillary Shoots

Elongated axillary shoots were individually separated and cultured onto MS medium containing 3% sucrose and supplemented with activated charcoal (0 and 1.5 mg/L) and IAA at 1 mg/L as an optimal auxin concentration for their rooting. The cultures were incubated at 25 ± 2 °C for 4 weeks under the dark followed by 3 weeks of light incubation or continuously incubated for 7 weeks under light (a 16 h photoperiod provided by cool white fluorescent lights at 35 μmol m^−2^·s^−1^ PPFD). The percentage of rooted plants, the number of roots per plant, length of roots, and fresh and dry weights were recorded. 

### 3.8. Acclimatization 

In vitro plantlets were removed from the medium and washed gently with sterile distilled water and then transferred to plastic pots containing a sterile mixture of peatmoss and perlite (1:1; *v*/*v*). The pots were covered with a transparent polyethylene plastic film for 4 weeks and kept in a growth chamber at 25 ± 2 °C, 50–60% RH, and 100 µmol m^−2^ s ^−1^ PPFD (16:8 h photoperiod under white fluorescent lamps). The cover was removed after 4 weeks. The plantlets were irrigated once a week with a nutrient solution containing ¼ strength of MS salts. Plantlet survival was evaluated 8 weeks after being transferred to the growth chamber. Finally, plantlets were cultured into pots containing a mixture of soil and peatmoss (1:1; *v*/*v*) and transferred to the greenhouse.

### 3.9. The Genetic Fidelity of F. palmata Micropropagated Plants

#### 3.9.1. Plant Materials and Genomic DNA Extraction

Ten micropropagated plantlets, two months old after transplantation, were randomly selected to test their genetic fidelity with the mother plant of *F. palmata.* The mother plant’s young, fresh leaves and those of the clonal regenerants were cleaned with sterile distilled water and stored in liquid nitrogen until molecular analysis. The leaf samples were ground to powder with liquid nitrogen using a mortar and pestle. The DNA was extracted using hexadecyltrimethyl-ammonium bromide (CTAB) method [100]. Approximately 100 mg of leaves was ground using 700 μL of preheated (65 °C) extraction buffer (2% CTAB, 20 mM EDTA, 100 mM Tris-HCl, 1.4 M NaCl, 1% polyvinylpyrrolidone (PVP), 0.2% mercaptoethanol), transferred to a centrifuge tube (2 mL), and incubated for 30 min in a 65 °C water bath. The samples were inverted every 5 min and 400 μL of chloroform-isoamyl alcohol (24:1) was added and mixed by inverting the tubes carefully. The cells were centrifuged at 12,000× *g* for 10 min at 4 °C. The supernatant was collected into new tubes containing 400 μL of chloroform-isoamyl alcohol (24:1) and the cells were centrifuged at 12,000× *g* for 10 min at 4 °C again. The supernatant was collected into new tubes and mixed with 600 μL ice-cold isopropanol, and the DNA samples were collected by centrifuging at 12,000× *g* for 10 min at 4 °C. Then, they were washed with ice-cold absolute and 500 μL ethanol 70% and centrifuged at 12,000× *g* for 5 min at 4 °C. Finally, the samples were dried at room temperature and dissolved in 50–100 μL of TE buffer. The quality and concentration of DNA were determined using a spectrophotometer (Jenway, Stafford, UK), and stored at −20 °C until use. 

#### 3.9.2. Randomly Amplified Polymorphic DNA (RAPD) Analysis

RAPD was carried out using ten primers (Table 6). The amplification reactions were performed in a 20 µL volume containing about 3 µL genomic DNA (50 ng/µL), 2 µL random primer and 10 µL of 2× green master mix (Promega, Madison, WA, USA), and 5 µL ultrapure (Milli-Q) water in thermal cycler machine. The PCR temperature profile was adjusted to 5 min at 94 °C for initial denaturation followed by 40 cycles of 1 min at 94 °C, an annealing step of 1 min at 37 °C for all primers except Primer (OPA-10) it was 35 °C (Table 6), and an elongation step of 1 min at 72 °C. Finally, a 7 min extension at 72 °C was applied. The amplified products were electrophoretically separated on a 2.5% agarose gel using 1× TBE buffer and stained with ethidium bromide (0.5 µg/ mL) and a 100 pb DNA ladder was used as the standard marker. The stained gels were scanned and photographed under the UVP DigiDoc-lt gel documentation system (Analytik Jena, UVP, Upland, CA, USA). Data were recorded as discrete variables: 1 for the presence and 0 for the absence of a similar band. Only intense and reproducible bands appearing on the gel were scored. 

#### 3.9.3. Inter Simple Sequence Repeats (ISSR) Analysis

ISSR was carried out using ten primers (Table 6); PCR for ISSR amplification was performed in a volume of 20 μL with the same concentrations and volumes of reaction components as for RAPD analysis. The PCR program consisted of an initial denaturation for 4 min at 94 °C, followed by 40 cycles of 1 min denaturation at 94 °C, an annealing step at 40–55 °C for one minute shown in Table 2, and 2 min extension at 72 °C, with a final extension at 72 °C for 10 min. After that, the same steps were taken in RAPD for amplified products.

#### 3.9.4. Start Codon Targeted (SCoT) Analysis

Ten primers were screened for SCoT amplification. PCR for SCoT amplification was performed in a volume of 20 μL with the same concentrations and volumes of reaction components as for RAPD analysis. The PCR program consisted of an initial denaturation for 5 min at 94 °C, followed by 38 cycles of 1 min denaturation at 94 °C, an annealing step for 0.45 s at 55–60 °C (Table 6), and a 72 °C elongation of 2 min. A concluding extension was tracked at 72 °C for 5 min. For amplified products, the same steps were taken as in RAPD. Each experiment was repeated three times to ensure accurate findings and to validate the repeatability of the RAPD, ISSR, and SCoT markers.

### 3.10. Grafting

Micropropagated *F. palmata* plants were utilized as rootstocks for grafting *F. carica* ‘Brown Turkey’. The rootstock plants were 2 years old (≈0.6 cm diameter) and were grown in large plastic pots (45 cm diameter) containing a mixture of sand and peatmoss (3:1; *v/v*). Top shoot scions were approximately 12–16 cm in length, and the leaves had been removed during their preparation. The leaves below the grafting point were kept to maintain the photosynthetic activity of the plants. Splice grafting was performed (Figure 5a–c) at a height of 30–50 cm from the root of the rootstock. After the grafting, the cambium between the rootstock and the scion was connected using an appropriate grafting tape (Buddy Tape), and the grafts were covered with clear plastic bags to maintain a high humidity and avoid tissue dehydration. Twelve weeks after grafting, the final percentages of graft survival were evaluated from nine grafts. 

### 3.11. Experimental Design and Data Analysis

The experiments were conducted in a completely randomized design. Each treatment contained five replicates and each replicate was represented by a magenta vessel containing 4 explants. Data were recorded using twelve randomly selected explants. The data were analyzed using analysis of variance (ANOVA), Tukey’s multiple range test, and Student’s unpaired *t*-test. The mean values were compared at *p* ≤ 0.05–0.001 in SAS (version 9.4; SAS Institute, Inc., Cary, NC, USA).

## 4. Conclusions

The highest axillary shoot multiplication of *F. palmata* was obtained on a medium containing 2 mg/L BAP. Among different cytokinins tested, BAP proved more effective than TDZ and zeatin. Dark incubation proved to be useful for shoot elongation and producing 100% in vitro rooting of shoots cultured onto a medium containing 1 mg/L IAA and 1.5 g/L activated charcoal. RAPD, ISSR, and SCoT molecular markers revealed a high degree of genetic fidelity (95.9%) of micropropagated *F. palmata* clones. Additionally, these plants proved compatible rootstock for *F. carica* ‘Brown Turkey’. Therefore, the established micropropagation protocol in this study can successfully be employed for commercial applications.

## Figures and Tables

**Table 1:** The effect of cytokinin concentrations on axillary shoot multiplication of *Ficus palmata* using nodal explants after 7 weeks in culture (5 weeks in MS medium supplemented with cytokinins followed by 2 weeks in MS medium without PGRs).

Treatments		Number of Shoots/Explant	Length of the Longest Shoot/Explant (cm)	Fresh Weight/Explant (g)	Dry Weights/Explant (g)
Type of cytokinin					
BAP		7.4 a	1.9 a	2.456 b	0.302 a
TDZ		8.5 a	1.1 b	4.167 a	0.351 a
Zeatin		3.1 b	1.3 b	0.716 c	0.143 b
*p*-value		<0.0001 ***	<0.0001 ***	<0.0001 ***	<0.0001 ***
Concentrations (mg/L)					
Control (C0)		1.4 d	1.1 b	0.142 c	0.035 c
C1		4.4 c	1.3 ab	1.941 b	0.215 b
C2		7.3 a	1.6 a	2.621 a	0.283 a
C3		7.5 a	1.6 a	2.755 a	0.300 a
C4		6.0 b	1.3 ab	2.470 a	0.263 ab
*p*-value		<0.0001 ***	0.027 *	<0.0001 ***	<0.0001 ***
Type of cytokinin × concentration (mg/L)					
Control	C0	1.4 e	1.2 def	0.142 d	0.035 d
BAP	C1	3.2 de	1.7 bc	0.957 d	0.185 b
	C2	7.4 c	2.3 a	2.226 c	0.305 a
	C3	11.2 a	1.9 ab	3.641 ab	0.389 a
	C4	7.7 c	1.7 bc	3.001 bc	0.331 a
TDZ	C1	8.5 bc	1.4 bcde	4.601 a	0.378 a
	C2	9.8 ab	1.0 ef	4.658 a	0.364 a
	C3	8.0 bc	1.0 ef	3.646 b	0.328 a
	C4	7.5 c	1.0 ef	3.765 ab	0.334 a
Zeatin	C1	1.5 e	0.7 f	0.265 d	0.080 cd
	C2	4.8 d	1.5 bcde	0.978 d	0.182 b
	C3	3.3 de	1.8 ab	0.977 d	0.183 b
	C4	2.8 e	1.3 cde	0.646 d	0.126 bc
*p*-value		<0.0001 ***	0.0001 ***	< 0.0001 ***	0.0001 ***

BAP concentrations (0.0, 0.1, 1.0, 2.0, and 3.0 mg/L), TDZ concentrations (0.0, 0.1, 0.5, 1.0, and 1.5 mg/L), and zeatin concentrations (0.0, 0.1, 0.5, 1.0, and 1.5 mg/L). Values followed by the same letter in each column are not significantly different at *p* ≤ 0.05 according to Tukey’s multiple range test. * and *** = significant at *p* ≤ 0.05 and *p* ≤ 0.001.

**Table 2:** The effect of light intensity and sucrose concentration on axillary shoot multiplication of *Ficus palmata* from nodal explants after 7 weeks in culture (5 weeks in MS medium supplemented with 2 mg/L BA followed by 2 weeks in MS medium without PGRs).

Treatments		Number of Shoots/Explant	Length of the Longest Shoot/Explant (cm)	Fresh Weight/Explant (g)	Dry Weights/Explant (g)
Light intensity (PPFD; μmol m^−2^·s^−1^)					
15		5.1 b	6.1 a	3.275 ab	0.337 a
35		7.2 a	6.3 a	3.791 a	0.349 a
70		3.7 b	5.1 b	2.376 b	0.321 a
*p*-value		<0.0001 ***	<0.0001 ***	<0.0001 ***	0.057 ^NS^
Sucrose (%)					
1.5		6.6 ab	6.8 a	3.752 ab	0.330 b
3.0		7.1 a	6.2 ab	4.346 a	0.420 a
4.5		5.1 b	5.7 b	3.139 b	0.382 ab
6.0		2.7 c	4.5 c	1.352 c	0.213 c
*p*-value		<0.0001 ***	<0.0001 ***	<0.0001 ***	<0.0001 ***
Light intensity × sucrose (%)					
15	1.5	6.6 bc	7.0 a	3.965 b	0.273 f
	3.0	6.0 c	6.0 bc	3.669 b	0.345 d
	4.5	4.5 d	6.9 ab	3.525 b	0.439 b
	6.0	3.6 de	4.7 ef	1.944 d	0.295 ef
35	1.5	7.4 b	6.7 ab	3.698 b	0.340 de
	3.0	10.9 a	7.5 a	6.659 a	0.514 a
	4.5	7.5 b	5.7 cd	3.562 b	0.348 d
	6.0	3.0 e	5.1 de	1.245 e	0.197 g
70	1.5	5.8 c	6.7 ab	3.593 b	0.377 cd
	3.0	4.3 d	5.1 de	2.710 c	0.401 bc
	4.5	3.2 e	4.6 ef	2.332 cd	0.358 cd
	6.0	1.4 f	3.9 f	0.868 e	0.149 h
*p*-values		<0.0001 ***	0.001 ***	<0.0001 ***	<0.0001 ***

Values followed by the same letter in each column are not significantly different at *p* ≤ 0.05 according to Tukey’s multiple range test. NS and *** = non-significant and significant at *p* ≤ 0.001.

**Table 3:** The effect of auxin concentration on in vitro rooting of *Ficus palmata* microshoots after 7 weeks in culture.

Treatments		Rooting (%)	Number of Roots/Explant	Length of Root (cm)	Fresh Weight/Explant (g)	Dry Weight/Explant (g)
Type of auxin						
IBA		35.0 b	1.0 a	0.9 b	0.852 b	0.204 b
NAA		30.0 b	0.8 a	0.5 b	1.510 a	0.263 a
IAA		50.0 a	1.3 a	2.3 a	0.848 b	0.208 b
*p*-value		0.011 *	0.520 ^NS^	0.002 **	0.001 ***	0.007 **
Concentration (mg/L)						
Control		41.6 a	1.2 ab	2.8 a	0.937 a	0.223 ab
0.1		41.6 a	1.8 a	1.7 abc	0.928 a	0.211 b
0.5		38.8 a	0.5 b	0.6 c	0.964 a	0.208 b
1.0		50.0 a	1.1 ab	1.8 ab	1.208 a	0.257 a
2.0		19.4 b	0.6 b	0.8 bc	1.179 a	0.223 ab
*p*-value		0.013 *	0.028 *	0.001 ***	0.109 ^NS^	0.168 ^NS^
Type of auxin × Concentration (mg/L)						
Control	0.0	41.7 b	1.2 abcd	2.8 ab	0.937 cde	0.223 abc
IBA	0.1	33.3 b	2.1 ab	1.6 bcd	0.754 de	0.192 bc
	0.5	33.3 b	0.7 bcd	0.2 d	0.726 e	0.179 c
	1.0	41.7 b	0.8 abcd	1.1 bcd	0.961 cde	0.233 abc
	2.0	25.0 bc	0.4 cd	0.8 cd	0.967 cde	0.212 abc
NAA	0.1	41.7 b	2.3 a	1.1 bcd	1.239 bcd	0.243 abc
	0.5	33.3 b	0.1 d	0.3 d	1.352 bc	0.248 abc
	1.0	33.3 b	0.8 abcd	0.5 cd	1.545 ab	0.284 a
	2.0	0.0 c	0.0 d	0.0 d	1.903 a	0.278 a
IAA	0.1	50.0 ab	1.2 abcd	2.4 abc	0.791 de	0.200 bc
	0.5	50.0 ab	0.8 abcd	1.4 bcd	0.815 de	0.198 bc
	1.0	75.0 a	1.8 abc	3.9 a	1.119 bcde	0.255 ab
	2.0	33.3 b	1.3 abcd	1.5 bcd	0.667 e	0.178 c
*p*-value		0.456 ^NS^	0.529 ^NS^	0.449 ^NS^	0.059 ^NS^	0.753 ^NS^

Values followed by the same letter in each column are not significantly different at *p* ≤ 0.05 according to Tukey’s multiple range test. NS, *, **, and *** = non-significant, and significant at *p* ≤ 0.05, *p* ≤ 0.01, and *p* ≤ 0.001, respectively.

**Table 4:** Effect of photoperiod and activated charcoal on rooting of *Ficus palmata* from microshoots after 7 weeks in culture in MS medium supplemented with 1 mg/L IAA.

Treatments		Rooting (%)	Number of Roots/Explant	Length of Root (cm)	Fresh Weight of Roots/Explant (g)	Dry Weight of Roots/Explant (g)
Photoperiod (h)						
Lightening regime 1		62.5	3.67	4.58	0.507	0.054
Lightening regime 2		100	5.75	2.93	0.101	0.019
*p*-value		0.006 **	0.018 *	0.021 *	0.001 ***	0.008 **
Activated charcoal (mg/L)						
0.0		83.3	4.29	3.43	0.363	0.043
1.5		79.1	5.13	4.08	0.245	0.030
*p*-value		0.347 ^NS^	0.088 ^NS^	0.124 ^NS^	0.272 ^NS^	0.375 ^NS^
Photoperiod (h) × Activated charcoal (mg/L)						
Lightening regime 1	0.0	67 b	3.67 b	3.71 ab	0.752 a	0.071 a
	1.5	75 b	3.83 b	6.21 a	0.518 a	0.058 ab
Lightening regime 2	0.0	100 a	4.67 b	3.24 b	0.123 b	0.026 bc
	1.5	100 a	6.67 a	3.0 b	0.087 b	0.014 c
*p*-value		0.347 ^NS^	0.147 ^NS^	0.063 ^NS^	0.417 ^NS^	0.956 ^NS^

Lightening regime 1 (Light/dark (16:8 h; 7 weeks) and lightening regime 2 (Light/dark (0:24 h) for 4 weeks followed by light/dark (16:8 h) for 3 weeks). Values followed by the same letter in each column are not significantly different at *p* ≤ 0.05 according to Tukey’s multiple range test. NS, *, **, and *** = non-significant, and significant at *p* ≤ 0.05, *p* ≤ 0.01, and *p* ≤ 0.001, respectively.

**Table 5:** Polymorphism percentage of micropropagated *F. palmata* plantlets obtained with RAPD, ISSR, and SCoT primers.

Primers	Primer Sequence 5′-3′	NO. of Scorble Band per Primer	Range of Amplification (bp)	No. of Monomorphic Bands	No. of Polymorphic Bands	Polymorphism %
RAPD primers						
OPA-01	CAGGCCCTTC	4	500–1000	4	0	0
OPA-03	AGTCAGCCAC	1	1300–1500	1	0	0
OPA-07	GAAACGGGTG	Nil	-	-	-	-
OPA-10	GTGATCGCAG	4	400–1000	4	0	0
OPB-03	CATCCCCCTG	2	200–600	2	0	0
OPH-05	AGTCGTCCCC	4	300–800	4	0	0
OPA-11	CAATCGCCGT	2	900–1500	2	0	0
OPA-12	TCGGCGATAG	3	250–1500	3	0	0
OPA-13	CAGCACCCAC	2	300–800	2	0	0
OPA-15	TTCCGAACCC	Nil	-	-	-	-
Total		22		22		0
ISSR primers						
ISSR-11	AGAGAGAGAGAGAGAGT	8	200–1500	8	0	0
ISSR-15	GAGAGAGAGAGAGAGAC	5	250–700	5	0	0
UBC815	CTCTCTCTCTCTCTCTG	3	300–900	3	0	0
UBC816	CACACACACACACACAT	5	400–800	5	0	0
UBC823	TCTCTCTCTCTCTCTCC	2	500–1200	2	0	0
UBC861	ACCACCACCACCACCACC	5	300–1000	5	0	0
UBC862	AGCAGCAGCAGCAGCAGC	3	350–700	3	0	0
IMA-5-Z	CACACACACACACACAGT	8	300–1500	6	2	25
IMA-5-3	CACACACACACACACATG	5	250–1000	5	0	0
HB15	GTGGTGGTGGC	6	400–1250	6	0	0
Total		50		48	2	4
SCoT primers						
S1	CAACAATGGCTACCACCA	4	250–800	4	0	0
S2	CAACAATGGTACCACCC	1	1500	1	0	0
S3	AACAATGGCTACCACCG	5	300–1400	5	0	0
S4	CAACAATGGCTACCACCT	4	350–800	4	0	0
S5	CAACAATGGCTACCACGA	Nil	-	-	-	-
S6	CAACAATGGCTACCACGC	Nil	-	-	-	-
S7	AGCAGCAGCAGCAGCAGC	6	300–900	4	2	33.3
S8	ACGACATGGCGACCAACG	4	300–1400	4	0	0
S9	ACCATGGCTACCACCGAC	2	800–1000	2	0	0
S10	CCATGGCTACCACCGCAG	4	400–1400	4	0	0
Total		30		28	2	6.6

**Table 6:** List of RAPD, ISSR, and SCoT primers used for testing the genetic fidelity of *F. palmata* micropropagated plants.

Primer No.	Primer Name	Primer Sequence	Annealing Temperature
		RAPD primers	
1	OPA-01	CAGGCCCTTC	37
2	OPA-03	AGTCAGCCAC	37
3	OPA-07	GAAACGGGTG	37
4	OPA-10	GTGATCGCAG	35
5	OPB-03	CATCCCCCTG	37
6	OPH-05	AGTCGTCCCC	37
7	OPA-11	CAATCGCCGT	37
8	OPA-12	TCGGCGATAG	37
9	OPA-13	CAGCACCCAC	37
10	OPA-15	TTCCGAACCC	37
ISSR primers			
1	ISSR-11	AGAGAGAGAGAGAGAGT	50
2	ISSR-15	GAGAGAGAGAGAGAGAC	50
3	UBC815	CTCTCTCTCTCTCTCTG	50
4	UBC816	CACACACACACACACAT	50
5	UBC823	TCTCTCTCTCTCTCTCC	50
6	UBC861	ACCACCACCACCACCACC	55
7	UBC862	AGCAGCAGCAGCAGCAGC	55
8	IMA-5-Z	CACACACACACACACAGT	50
9	IMA-5-3	CACACACACACACACATG	50
10	HB15	GTGGTGGTGGC	40
SCoT primers			
1	S1	CAACAATGGCTACCACCA	55
2	S2	CAACAATGGTACCACCC	55
3	S3	AACAATGGCTACCACCG	56
4	S4	CAACAATGGCTACCACCT	56
5	S5	CAACAATGGCTACCACGA	55
6	S6	CAACAATGGCTACCACGC	55
7	S7	AGCAGCAGCAGCAGCAGC	57
8	S8	ACGACATGGCGACCAACG	55
9	S9	ACCATGGCTACCACCGAC	55
10	S10	CCATGGCTACCACCGCAG	60

## Data Availability

All data are presented in the article.
